# Association between eveningness and depressive symptoms in daytime workers: A cross-sectional analysis of the Aichi Workers’ Cohort Study

**DOI:** 10.20407/fmj.2024-021

**Published:** 2024-12-27

**Authors:** Kazuhito Ishihara, Tsuyoshi Kitajima, Atsuhiko Ota, Hiroshi Yatsuya, Nakao Iwata

**Affiliations:** 1 Department of Psychiatry, Fujita Health University, School of Medicine, Toyoake, Aichi, Japan; 2 Department of Public Health, Fujita Health University, School of Medicine, Toyoake, Aichi, Japan; 3 Department of Public Health and Health System, Nagoya University Graduate School of Medicine, Nagoya, Aichi, Japan

**Keywords:** Chronotype, Eveningness, Depression, Daytime workers, Cross-sectional study

## Abstract

**Objectives:**

Eveningness (evening preference in chronotype) has been reported to be associated with a number of psychiatric problems. We examined a cross-sectional association between eveningness and depressive symptoms in daytime workers.

**Methods:**

The subjects were 4410 civil servants (71.7% male, aged 18–69 years [mean, 43.5 years]) with no history of depressive disorder who did not perform shift work, and for whom there were no missing data. The association between eveningness, determined using the reduced Morningness–Eveningness Questionnaire, and depressive symptoms, determined using the shorter form of the Central Epidemiological Studies Depression scale, was assessed with logistic regression analysis adjusted for potential confounding factors (gender, income, living alone, breakfast intake, coffee consumption, drinking habits, exercise, smoking, working hours, sleep duration, and difficulty falling asleep).

**Results:**

Of the subjects, 529 (12.0%) had eveningness, and 1408 (31.9%) reported depressive symptoms. Depressive symptoms were reported more frequently by those with eveningness than by those with other chronotypes (57.3% vs 28.5%). The association between eveningness and depression (gender- and age-adjusted odds ratio, 3.27; 95% confidence interval, 2.71–3.95) was significant after adjustment for the other confounding factors (odds ratio, 2.40; 95% confidence interval, 1.96–2.95).

**Conclusions:**

Our results suggest that eveningness is associated with depressive symptoms among day workers and that this association is independent of potential confounding factors. Further longitudinal studies are needed to examine the causal relationship.

## Introduction

Depressive disorder is characterized by symptoms such as depressed mood, loss of interest, lack of motivation, inability to sleep, fatigue, and tiredness, all of which can affect work functionality. A cross-national analysis of population surveys from 29 countries, including low- to high-income nations, revealed a lifetime prevalence of major depressive disorder of 7.5% among men and 13.6% among women, with most cases occurring in those of working age.^1^ Epidemiological studies from Japan and Australia showed that depression is associated with both absenteeism and presenteeism in the workplace.^2,3^ Therefore, preventing depression is crucial for maintaining workers’ mental health.

Nearly all species exhibit internal and biological circadian rhythms that last approximately 24 hours. Their alignment with the environmental light/dark cycle is believed to be important for biological functions in humans. In mood disorders such as depression, the sleep–wake rhythm is often disrupted, which sometimes leads to circadian rhythm sleep–wake disorders.^4,5^ The recurrence of mood episodes in bipolar disorder is also associated with circadian rhythm disturbances.^6,7^ By affecting the circadian rhythms, light therapy can alleviate mood symptoms.^8^ Basic research has indicated a link between circadian rhythm mechanisms, including clock genes, and mood regulation.^9,10^ Thus, depression and other mood disorders are strongly related to circadian rhythms.

In recent years, chronotype has been used as an indicator of human circadian rhythm preferences. Chronotype is assessed with questionnaires such as Horne and Östberg’s Morningness–Eveningness Questionnaire (MEQ).^11^ Chronotypes have a normal distribution among the general population^12^ and are determined by genetic and environmental factors.^13^ According to many studies, including meta-analyses, eveningness is associated with various psychiatric conditions, especially depression.^14,15^ In contrast, behaviors related to depressive tendencies may also change the sleep–wake rhythm to a nocturnal pattern. Eveningness and depression are thought to share the relevant factors, such as sleep duration,^16–18^ breakfast intake,^19–21^ smoking,^22–24^ alcohol intake,^25–28^ physical activity,^29,30^ difficulty falling asleep,^31^ working hours,^32^ living alone,^33,34^ coffee consumption,^35,36^ and income.^37^ Most studies of the association between depression and chronotype have focused on students, the general population, and psychiatric outpatients. Some of those individuals, however, may not have time-restrictive schedules.

In this study, we examined the association between chronotype and depressive symptoms in a cohort study of only daytime workers. We were able to analyze many of the potential confounding factors described previously that are reportedly associated with both eveningness and depression.

## Methods

### Subjects

A cross-sectional study was conducted as part of the Aichi Workers’ Cohort Study,^38–40^ a prospective investigation conducted among public employees. Between July and August 2018, we distributed a survey to these civil servants, 5543 of whom responded (3715 [67%] were men). We excluded 167 respondents with a history of treatment for depressive disorder, 800 who performed rotating shift work or worked night shifts (of whom 18 also had a history of treatment for depressive disorder), and 184 for whom data were missing; the data of the remaining 4410 employees were analyzed.

This study was approved by the Ethical Review Boards of Nagoya University School of Medicine, Nagoya, Japan (approval number 2013-0005), and Fujita Health University, Aichi, Japan (approval number HM20-048). All participants gave written consent.

### Measurements

#### Chronotype

To assess chronotype, we administered the reduced Morningness–Eveningness Questionnaire (rMEQ) to the participants.^41^ The rMEQ is a shortened five-item version of the MEQ,^11^ and has been validated for reliability and validity.^42^ Scores of 22–25 represent “definitely morning type;” 18–21, “moderately morning type;” 12–17, “neither type;” 8–11, “moderately evening type;” and 4–7, “definitely evening type.” For this study, the last two were combined into an “eveningness” category, and the first three were combined into one category, the values of which were used as references.

#### Depressive symptoms

To assess depressive symptoms, we used a short form of the Center for Epidemiologic Studies Depression Scale (CES-D).^43,44^ The original CES-D, a 20-item self-rating scale developed by the Center for Epidemiologic Studies of the National Institute of Mental Health, is a widely used psychological test for assessing depressive symptoms. The version used in this study, which has been tested for reliability and validity, consists of 11 questions about the frequency of symptoms per week; each response is scored on a 4-point scale from 0 to 3. In our study, 8 points or more^43–45^ indicated the presence of depressive symptoms.

#### Covariates

The following potential confounding factors served as covariates because of their reported or supposed association with both eveningness and depression: sleep duration,^16–18^ breakfast intake habits,^19–21^ drinking habits,^25–28^ exercise habits,^29,30^ smoking habits,^22–24^ difficulty falling asleep,^31^ working hours,^32^ living alone,^33,34^ coffee consumption,^35,36^ and income.^37^

### Statistical analysis

The associations between the aforementioned covariates and eveningness were confirmed using logistic regression analysis, in which all covariates, gender, and age were simultaneously entered as independent variables. The association between eveningness and depressive symptoms was analyzed with logistic regression models adjusted for gender and age (model 1) and adjusted for gender, age, and the ten covariates (model 2). In these analyses, all data except for age and working hours were dichotomized and used as covariates because of the robustness of the analysis for their distributions. The cut-offs (shown in italics) for the dichotomizations were employed as follows: *Breakfast consumption twice a week or less*, which was reported to be associated with evening chronotype^19^; *sleep duration of 5 hours or less*, which is contrary to the recommendations for adults in the latest Japanese health guidelines^46^; *drinking habits of three or more times a week*, which is from the definition of a habitual drinker in a Japanese national survey^47^; *exercise habits of less than three times a week*, which was associated with the incidence of type 2 diabetes in a previous study of the same cohort^48^; *smoking cigarettes currently, with a lifetime history of having smoked at least 100 cigarettes*^49^ was defined as current smoking as per a national survey in the United States; *difficulty falling asleep three times a week or more* as described in the diagnostic criteria for insomnia in the International Classification of Sleep Disorders^50^; *coffee consumption of two or more cups a day*, based on a report that the consumption of two or three cups of coffee was associated with a low risk of depression or anxiety^35^; *an annual income of JPY 6 million or more* was taken from the average income in Japan according to a recent national survey.^51^ Working hours were divided into four categories (4 hours or less, 5–8 hours, 9–12 hours, and 13 or more hours a day) because of the hypothetical direct dose-dependent association with both eveningness and depression; values in the 5–8 hours of work category were used as the references. Age served as a continuous variable.

We performed a sensitivity analysis stratified by gender and age (≤39 years and ≥40 years) as described previously because of chronotypes being distributed differently by these variables.^52^

We used IBM SPSS version 28 (IBM Corporation, Armonk, NY, USA) for all analyses.

## Results

The characteristics of the 4410 subjects are listed in Table 1. Participants’ ages ranged from 18 to 69 years (mean 43.5 years); 64.8% were aged 40 or older, and 71.7% were male. On the rMEQ, eveningness scores characterized 12.0% of all the subjects, of whom 71.8% were male; 18.3% of those aged 39 or younger, of whom 69.1% were male; and 8.5% of those aged 40 or older, of whom 75.0% were male. Depressive symptoms characterized 36.0% of subjects aged 39 years and younger, of whom 60.6% were male, and 29.7% aged 40 years and older, of whom 71.5% were male.

Eveningness was significantly associated with short sleep, skipping breakfast, less frequent drinking, less frequent exercise, current smoking, difficulty falling asleep, moderately more working hours, and living alone, but not with less working hours, extremely more working hours, coffee consumption, and income (Table 2). The frequency of depressive symptoms was higher among subjects with the eveningness chronotype (57.3%) than among those with morningness or neither chronotype (28.5%). The analysis adjusted for age and gender (model 1) revealed a significant association between eveningness and depressive symptoms (odds ratio, 3.27; 95% confidence interval, 2.71–3.95). The association remained significant after adjusting for sleep duration, breakfast habits, drinking habits, exercise habits, smoking history, difficulty falling asleep, working hours, living alone, coffee consumption, and income (model 2; odds ratio, 2.40; 95% confidence interval, 1.96–2.95; Table 3).

In the analysis stratified by age, of the 968 men and 584 women aged 39 years or younger, 197 (20.4%) men and 88 (15.1%) women showed eveningness, and 339 (35.0%) men and 220 (37.7%) women exhibited depressive symptoms. Of the 2195 men and 663 women aged 40 years or older, 83 (8.3%) men and 61 (9.2%) women displayed eveningness, and 607 (27.7%) men and 242 (36.5%) women had depressive symptoms; among women in that age group, the association between eveningness and depressive symptoms did not reach statistical significance after adjustment (Table 3). Among men, the association between eveningness and depressive symptoms was significant for both those aged 39 years or younger and those aged 40 years or older (Table 3).

## Discussion

This study found that the association between eveningness and depressive symptoms among daytime workers was independent of potential confounding factors. A previous meta-analysis revealed a significant association between eveningness and depression.^14,15^ However, in many previous studies, the nocturnal patterns of a certain percentage of individuals were further enhanced by behavioral factors, such as excessive media use or bedtime procrastination, as a result of their relatively less restrictive time schedules. Our participants were adults of a wide range of ages who all lived according to their work schedules. Therefore, we considered that the influences of poor social constraints or social adjustment status, which are common among the typical participants in such studies, such as students, the general population, and psychiatric outpatients, would be small. The relatively few studies that have focused on workers ranging in age from young to middle-aged with regular work schedules include an online survey of 3917 men and women by Kim et al.,^53^ an assessment of 950 factory-working men by Furusawa et al.,^54^ and an assessment of 202 women by Haraszti et al.^55^ All of these studies revealed a significant association between eveningness and depression. We examined a larger population and adjusted for the potential confounding factors that are supposedly associated with both eveningness and depression. In fact, although almost all of these factors were associated with eveningness (other than the negative association with alcohol habits and the lack of association with less working hours, extremely more working hours, coffee consumption, and income), the majority were also associated with depressive symptoms independently of eveningness (data not shown); nevertheless, to our knowledge, this is the first study to demonstrate that eveningness is associated with depressive symptoms independently of these factors.

In this study, workers with a history of treatment for depressive disorder and those performing shift work were excluded. In workers with a history of treatment for depression, it is possible that stress or conditions other than chronotype were the main factors contributing to the onset of depression. In addition, although previous studies have shown an association between chronotype and depression among shift workers,^56,57^ additional factors, such as a misalignment between the working hours of shift workers and their chronotypes, of shift workers may lead to depressive tendencies. By excluding these factors from this study, we believe we were able to demonstrate more clearly the association between eveningness and depressive symptoms in the context of living on a regular day/night schedule.

### Effects of age and gender on eveningness and depressive symptoms

In sensitivity analyses stratified by age and gender, the association was not statistically significant for women aged 40 or older. Eveningness is reportedly present to a lesser degree in older age and in women,^52^ and in this study, the prevalence of eveningness among older women, 9.2%, was lower than the overall rate of 12.0%. Despite the large total number of women in this category, the low percentage of eveningness might make it difficult to find a statistically significant association with depressive symptoms; i.e., it cannot be ruled out that there was a lack of statistical power for this category alone. It is also possible that factors related to life events that are relatively common among women, including marriage, childbirth, and childrearing, may be involved. However, we could not clarify the reasons for this in this study; therefore, further investigation is needed.

### Mechanisms of association between eveningness and depressive symptoms

As this was a cross-sectional study, the causal relationship between eveningness and depressive symptoms is unclear. However, the association could be, for example, related to workers with eveningness needing to work during the day when their capacity for activity is reduced; this could lead to lower work performance and additional stress, which may also lead to lower self- and peer esteem. Additionally, as pointed out in cases of social jet lag, the mismatch between the internal circadian rhythms and the external schedule may be related to a decline in physical and mental functioning.^58^ Conversely, depressive symptoms, to the extent that they do not lead to a major depressive episode, may fluctuate diurnally, with a sluggish morning and a pickup in the afternoon, which increases the degree of eveningness. However, the results of many investigations, including basic studies, have indicated that circadian rhythm regulation mechanisms, including clock genes, might have the same biological basis for both eveningness and depression or mood disorders.^10^ Further studies are needed to examine these mechanisms in combination.

### Strengths and limitations of the study

The strengths of this study include its very large study population and careful consideration of potential confounding factors compared with previous reports. As a result, the study revealed a robust association between eveningness and depressive symptoms. However, the possibility that the lack of a significant association for women aged 40 or older might be due to a lack of statistical power for this subcategory cannot be ruled out. The limitations include the following. The chronotype and depressive symptoms were assessed using shortened versions of the respective questionnaires, which may be slightly less accurate than the full versions. The assessment of sleep was limited because we relied on a small number of questions, did not examine sleep quality, and did not collect data reflecting the objectivity of an actigraph or similar instrument. We also did not categorize aspects of breakfast, alcohol, exercise, and smoking in terms of quantity or content. Finally, because this was a cross-sectional study, no clear causal relationship between nocturnal sleep and depressive symptoms remains unknown, and further studies, including longitudinal ones, are needed.

## Conclusions

Our results suggest that eveningness is associated with depressive symptoms in daytime workers and that this association is independent of sleep duration, breakfast habits, drinking habits, exercise habits, smoking habits, difficulty falling asleep, working hours, living alone, coffee consumption, or income. The causal relationship is unclear because this was a cross-sectional study, and further studies are needed to confirm our findings.

## Figures and Tables

**Table1:** Characteristics of subjects: Aichi Workers’ Cohort Study, 2018

Factor	All	Eveningness chronotype	Morningness or neither chronotype
n	4410	529	3881
Age, mean (SD)	43.5 (11.6)	38.5 (11.5)	44.23 (11.5)
Gender (male)	3163 (71.7%)	380 (71.8%)	2783 (71.7%)
With depressive symptoms^a^ (+)	1408 (31.9%)	303 (57.3%)	1105 (28.5%)
Short sleep (<6 hours)	664 (15.1%)	147 (27.8%)	517 (13.3%)
Breakfast consumption (<3 times/week)	408 (9.3%)	117 (22.1%)	291 (7.5%)
Alcohol drinking (≥3 times/week)	1448 (32.8%)	120 (22.7%)	1328 (34.2%)
Exercise (<3 times/week)	3003 (68.1%)	398 (75.2%)	2605 (67.1%)
Current smoking (+)	413 (9.4%)	64 (12.1%)	349 (9.0%)
Difficulty falling asleep (≥3 times/week)	365 (8.3%)	106 (20.0%)	259 (6.7%)
Working hours			
≤4 hours/day	55 (1.2%)	4 (0.80%)	51 (1.3%)
5–8 hours/day	2829 (64.1%)	284 (53.7%)	2545 (65.6%)
9–12 hours/day	1421 (32.2%)	222 (42.0%)	1199 (30.9%)
≥13 hours/day	105 (2.4%)	19 (3.6%)	86 (2.2%)
Living alone (+)	581 (13.2%)	132 (25.0%)	449 (11.6%)
Coffee consumption (≥2 cups/day)	1040 (23.6%)	117 (22.1%)	923 (23.8%)
Income (<JPY 6 million/year)	1219 (27.6%)	210 (39.7%)	1009 (26.0%)

^a^ Depressive symptoms were evaluated using a short version of the Center for Epidemiologic Studies Depression Scale (scores of ≥8 indicated the presence of such symptoms). SD: Standard deviation

**Table2:** Association between eveningness and potential confounding factors: Aichi Workers’ Cohort Study, 2018

Factor	Association with eveningness: odds ratio (95% confidence interval)		
	All subjects (n=4410)	Men (n=3163)	Women (n=1247)
Age (year)	0.96 (0.95–0.97)	0.95 (0.94–0.97)	0.97 (0.95–0.99)
Gender (male)*	1.26 (1.01–1.57)	—	—
Short sleep (<6 hours)	2.72 (2.15–3.44)	2.98 (2.26–3.94)	2.26 (1.45–3.53)
Breakfast consumption (<3 times/week)	2.70 (2.09–3.48)	2.28 (1.68–3.09)	3.23 (1.95–5.35)
Alcohol drinking (≥3 times/week)	0.76 (0.60–0.96)	0.82 (0.63–1.06)	0.61 (0.34–1.10)
Exercise (<3 times/week)	1.28 (1.02–1.59)	1.36 (1.05–1.76)	1.08 (0.70–1.67)
Current smoking (+)	1.39 (1.01–1.91)	1.47 (1.07–2.03)	0.16 (0.01–1.87)
Difficulty falling asleep (≥3 times/week)	2.86 (2.19–3.73)	2.89 (2.09–3.99)	2.84 (1.77–4.56)
Working hours			
≤4 hours/day	0.60 (0.21–1.74)	0.48 (0.11–2.13)	0.82 (0.18–3.75)
5–8 hours/day	Reference	Reference	Reference
9–12 hours/day	1.32 (1.08–1.62)	1.08 (0.85–1.38)	2.10 (1.44–3.07)
≥13 hours/day	1.49 (0.85–2.61)	1.67 (0.87–3.21)	1.06 (0.33–3.34)
Living alone (+)	1.70 (1.31–2.20)	1.63 (1.19–2.22)	1.82 (1.13–2.94)
Coffee consumption (≥2 cups/day)	1.10 (0.87–1.39)	1.19 (0.91–1.56)	0.84 (0.48–1.44)
Income (<JPY 6 million/year)	1.01 (0.79–1.29)	0.99 (0.74–1.32)	1.01 (0.63–1.61)

All variables listed in the table were simultaneously entered as independent variables. * Gender was not entered in gender-stratified analyses.

**Table3:** Associations between eveningness and depressive symptoms: Aichi Workers’ Cohort Study, 2018

Eveningness	Association with depressive symptoms: odds ratio (95% confidence interval)		
	All subjects (n=4410)	Men (n=3163)	Women (n=1247)
All ages	**(n=4410 [100%])**	**(n=3163 [71.7%])**	**(n=1247 [28.3%])**
Model 1	3.27 (2.71–3.95)	3.26 (2.61–4.08)	3.19 (2.23–4.55)
Model 2	2.40 (1.96–2.95)	2.49 (1.96–3.17)	2.15 (1.46–3.17)
≤39 years old	**(n=1552 [35.2%])**	**(n=968 [22.0%])**	**(n=584 [13.2%])**
Model 1	3.16 (2.42–4.12)	2.83 (2.06–3.91)	3.99 (2.46–6.46)
Model 2	2.47 (1.86–3.28)	2.31 (1.64–3.25)	2.92 (1.72–4.96)
≥40 years old	**(n=2858 [64.8%])**	**(n=2195 [49.8%])**	**(n=663 [15.0%])**
Model 1	3.36 (2.57–4.40)	3.72 (2.73–5.08)	2.37 (1.39–4.05)
Model 2	2.36 (1.76–3.17)	2.75 (1.96–3.86)	1.55 (0.85–2.81)

Model 1: Adjusted for gender and age. Only age was adjusted for in gender-stratified analyses. Model 2: Adjusted for gender, age, sleep, breakfast, alcohol, exercise, smoking, difficulty falling asleep, working hours, living alone, coffee, and income except for gender-stratified analyses, where gender was not included.
